# Supplementary material for: Balanced Translocations Involving the DMD Gene as a Cause of Muscular Dystrophy in Female Children: A Description of Three Cases
Source: Int J Mol Sci. 2025 Sep 25;26(19):9389. doi: 10.3390/ijms26199389 (PMC12525165; doi:10.3390/ijms26199389)
Supplement: Supplementary file 1 [file ijms-26-09389-s001.zip › ijms-3885879-supplementary.pdf]

Visualization of WGS reads in the IGV browser for all 3 patients.

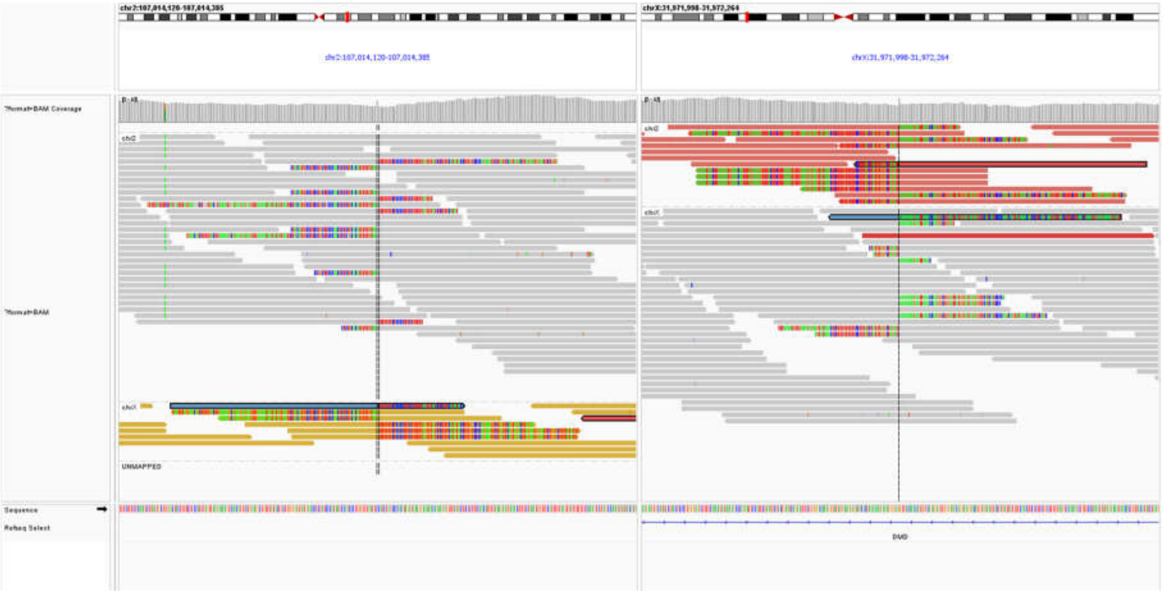

Figure S1. WGS reads in the IGV browser for Patient N1. A reciprocal translocation of short arm of chromosome X and long arm of chromosome 2 was identified. The breakpoint on the X chromosome is located in intron 1 of the DMD gene (NC_000023.11:g.pter_31972130delins[NC_000002.12:g.pter_107014253inv]), with a single nucleotide duplication chrX:31972131 in the breakpoint region. On chromosome 2, a breakpoint region NC_000002.12:g.107014254_qterdelins[TTTTTTT;NC_000023.11:g.pter_31972131inv] is accompanied by a single nucleotide duplication chr2:107014253 and non-template T_7-tract insertion.

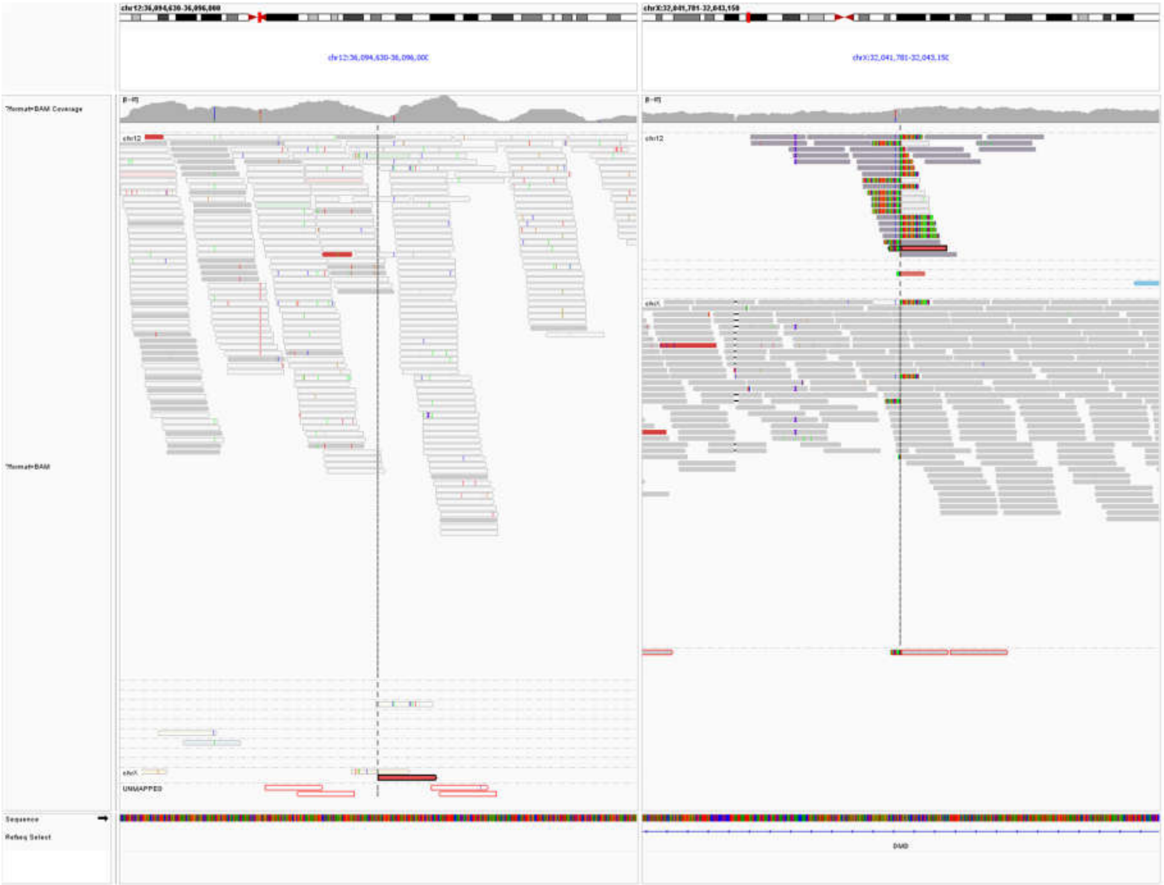

Figure S2. WGS reads in the IGV browser for Patient N2. A possible reciprocal translocation of short arm of chromosome X and long arm of chromosome 12 was identified. The breakpoint on the X chromosome is located in intron 44 of the DMD gene (NC_000023.11:g.pter_32042465delins[NC_000012.12:g.(q11-12)_qterinv]). It was not possible to determine the exact breakpoint on chromosome 12 due to the mapping of rearrangement-associated reads to an alpha-satellite repeat (NC_000012.12:g.(q11-q12)_qterdelins[NC_000023.11:g.32042468_qterinv]), which is typically localized in pericentromeric regions; no coding sequences were present in this region. The rearrangement also caused a dinucleotide deletion: NC_000023.11:g.32042466_32042467del.

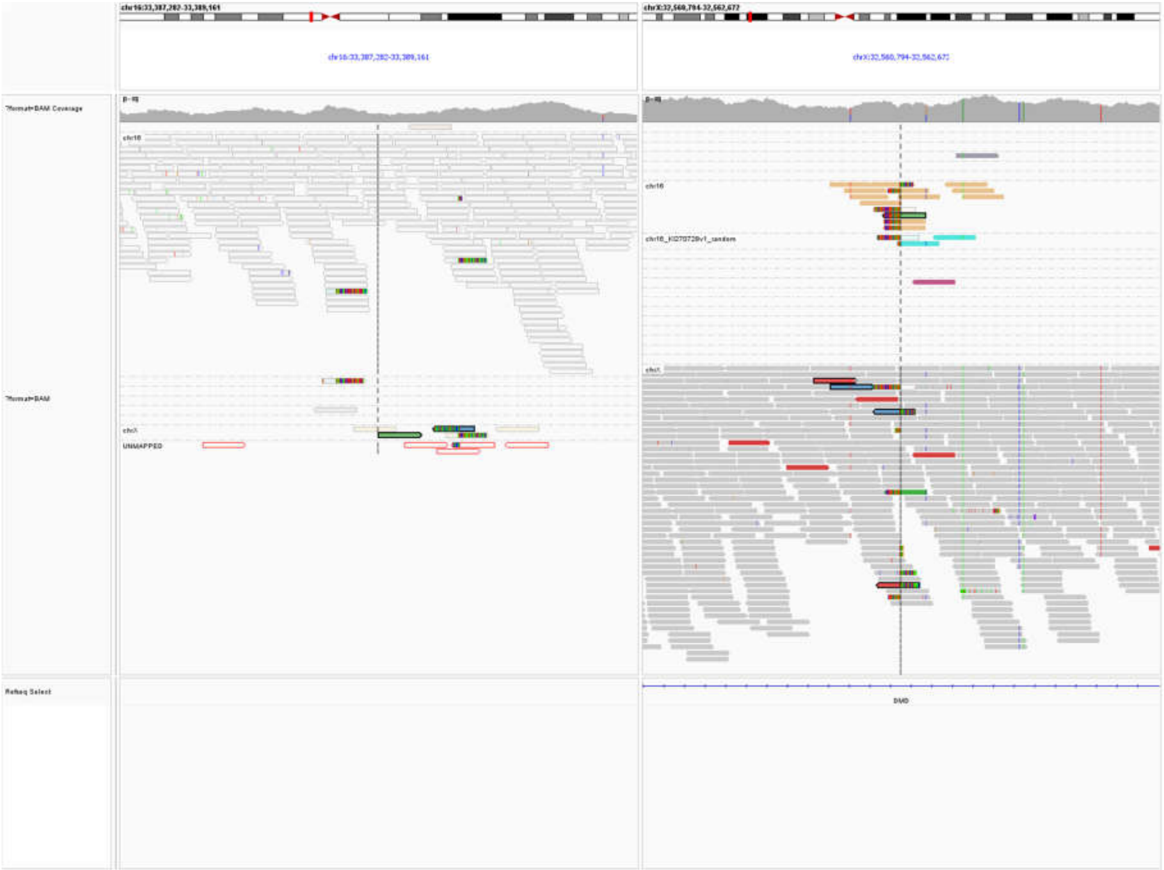

Figure S3. WGS reads in the IGV browser for Patient N3. A possible reciprocal translocation of short arm of chromosome X and either chromosomes 16 or 7 was detected. The breakpoint on the X chromosome is located within intron 16 of the DMD gene (NC_000023.11:g.pter_32561731delins[?]). The second breakpoint could not be precisely determined due to the mapping of rearrangement-associated reads to pericentromeric regions of several chromosomes, primarily chromosomes 16 and 7. The rearrangement also caused a single-nucleotide deletion: NC_000023.11:g.32561732del at the breakpoint site.
